# Synthesis and uranyl(VI) extraction performance of a calix[4]pyrrole–tetrahydroxamic acid receptor

**DOI:** 10.3762/bjoc.22.36

**Published:** 2026-03-18

**Authors:** Sara Karnib, Rana Baydoun, Wissam Zaidan, Nancy AlHaddad, Omar El Samad, Bilal Nsouli, Francine Cazier-Dennin, Pierre-Edouard Danjou

**Affiliations:** 1 Unité de Chimie Environnementale et Interactions sur le Vivant, UR 4492, Université du Littoral Côte d'Opale, 145 Avenue Maurice Schumann, MREI 1, Dunkerque, France; 2 Lebanese Atomic Energy Commission, National Council for Scientific Research CNRS –L, P.O. Box: 11-8281, Riad el soleh, 1107 2260, Beirut, Lebanon

**Keywords:** gamma spectroscopy, hydroxamic acid, phenoxycalix[4]pyrrole, solid–liquid extraction, uranyl(VI) extraction

## Abstract

The contamination of water by uranium poses a serious threat to ecosystems and human health, creating a need for efficient and selective remediation strategies. Supramolecular materials, with their pre-organized structures, offer a promising route for uranium removal. Phenoxycalix[4]pyrroles (PCP) are well-known supramolecular scaffolds capable of selective metal binding, making them attractive candidates for designing uranium extractants. Here, we report the design and synthesis of PCP HA, a phenoxycalix[4]pyrrole scaffold functionalized with four hydroxamic acid (HA) groups, and evaluate its uranium(VI) extraction potential. PCP HA was synthesized from its ester precursor (PCP E) via hydroxyaminolysis using KOH, achieving a 95% yield. Its structure was confirmed by ^1^H NMR, ^13^C NMR, and HRMS. The uranium(VI) extraction efficiency of PCP HA was evaluated by solid–liquid extraction experiments, using uranyl acetate as the uranium source, with measurements performed by gamma spectroscopy. PCP HA demonstrated good performance, removing up to 95% of uranyl(VI) from aqueous solutions (1 mM) at acidic pH, likely due to the strong coordination provided by its hydroxamic acid groups. Further studies revealed that the extraction efficiency also depends on the ligand-to-metal molar ratio. These findings establish PCP HA as a promising supramolecular material for the removal of uranyl from aqueous media.

## Introduction

Over the past few decades, supramolecular chemistry has advanced intensively establishing itself as a central discipline in modern chemistry [1–3]. This tremendous growth allowed the field to transcend the traditional chemistry boundaries and move toward real-life applications [4]. In this context, supramolecular materials with pre-organized structures have been designed and exploited for diverse environmental, industrial and biological applications [5–8].

Among these macrocycles, calix[4]pyrroles (CPs) represent a modern generation of supramolecular materials with interesting chelation properties [9]. These nonplanar, non-aromatic, tetrapyrrolic macrocycles consist of four pyrrolic units linked together at their 2,5-positions through sp^3^-hybridized *meso*-carbon atoms [10]. The ability of CPs to selectively bind anions [11], cations, ion pairs and neutral guest species, combined with the versatility to introduce diverse functional groups, has enabled the fabrication of calix[4]pyrrole-based supramolecular matrices for a broad range of applications [9]. Notable examples include catalysis, chromogenic sensors and fluorescent sensors, as well as heavy metals extraction [12–16].

One CP molecule that has attracted the attention of several research groups is the *meso*-tetra-methyltetrakis(4-hydroxyphenyl)calix[4]pyrrole, commonly referred to as phenoxycalix[4]pyrrole (PCP). It was first reported in 1999 by two independent research groups, Floriani et al. and Sessler et al., via the acid-catalyzed condensation of pyrrole with *p*-hydroxyacetophenone [17–18]. PCP and its derivatives were first known for their anion chelation capability achieved through the formation of hydrogen bonds with the pyrrolic units [19–22]. Subsequent studies have demonstrated that the introduction of additional chelation sites via extension of the phenolic groups of PCP can yield anionic receptors, ditopic receptors bearing both anion- and cation-binding sites, as well as systems capable of promoting ion-pair formation [23–24].

As effective chelating agents for a broad range of transition metals, hydroxamic acids constitute an important class of organic compounds that have attracted considerable attention and have found diverse applications across both biomedical and industrial fields [25–26]. Hydroxamic acids are also well known for their strong chelating ability toward uranyl, forming stable complexes through the synergistic coordination of the carbonyl and hydroxylamine groups [27]. Uranium is a naturally occurring radionuclide and is thus an integral constituent of the environment [28]. However, beyond its extensive use as fuel for nuclear power generation, uranium is released into the environment through various industrial processes, such as phosphate fertilizer production and metal refining, contributing to the increased accumulation of uranium in ecosystems [29–31]. Owing to its radioactivity, chemical toxicity, and long half-life, environmental uranium contamination poses serious ecological and public health risks [32]. In this context, hydroxamic acid functionalities have been successfully incorporated on supramolecular architectures to explore their potential for the removal of uranium from aqueous media. For instance, it has been long known that hydroxamic acid derivatives of calix[4]arenes and calix[6]arenes act as excellent uranophiles as demonstrated in early studies from 1991 [33]. A calix[4]resorcinarene hydroxamic acid has also shown a pronounced binding tendency and selectivity for uranyl and proved to be applicable for the determination of uranium in standard and environmental samples [34]. Importantly, more recent studies have demonstrated that pre-organized or cyclic hydroxamate ligands can display significantly enhanced binding toward uranyl relative to their linear hydroxamic acid counterparts, emphasizing the beneficial role of structural organization in uranyl chelation [35]. This beneficial role of pre-organization of chelating groups within one molecule was also highlighted for supramolecular platforms like calixarenes for actinides and lanthanides extraction [36–37].

In light of this background, we report here the incorporation of hydroxamic acid into phenoxycalix[4]pyrrole and examine its effectiveness in extracting uranyl from aqueous solution using gamma spectroscopy. The extraction performance was systematically evaluated as a function of pH and ligand-to-metal molar ratio to study the effect of these two key parameters on the extraction process. In doing so, this work aligns with current research dedicated to the design of functional supramolecular materials capable of remediating uranium from aqueous environments [38–39].

## Results and Discussion

### PCP HA synthesis and characterization

Various strategies have been developed for the synthesis of hydroxamic acid derivatives over the years [40]. Classical and most popular routes involve the direct reaction of carboxylic acid derivatives such as esters, acyl chlorides and anhydrides with hydroxylamine salts [41]. A variety of coupling or activating agents were also employed in case of simple addition of hydroxylamine to carboxylic acid compounds [42–44]. In addition, alternative methods starting from aldehydes [45], alcohols [46], and amides [47] have also been reported. However, the literature states that there is no particular reagent or reaction condition that can serve as a general rule for the synthesis of hydroxamic acids [48].

In this study, the direct reaction of hydroxylamine with the PCP ester was first examined, since the ester functions as the synthetic precursor to the corresponding acid used in the preparation of PCP derivatives from PCP [21]. For that, PCP was initially synthesized through the molecular cyclisation of pyrrole with 4-hydroxyacetophenone in methanol in the presence of methanesulfonic acid as first reported by Floriani et al. [17] and Sessler et al. [18]. This condensation reaction produces several PCP isomers, including the targeted pre-organized α,α,α,α-isomer, which is isolated by crystallization from glacial acetic acid, followed by removal of the acetic acid using a mixture of acetonitrile and acetone. Using this protocol, the PCP α,α,α,α-isomer was recovered in 47% yield, as previously reported by Namor and Shehab [49]. Subsequently, the synthesis of the PCP ester derivative (PCP E) was carried out via O-alkylation of PCP with ethyl bromoacetate in dry acetone/K_2_CO_3_, following the method reported by Camiolo and Gale [21], affording a yield of 85%.

Among several procedures used for the direct hydroxyaminolysis of carboxylic ester substrates, Beillard et al. highlighted the superiority of a DBU (1,8-diazabicyclo[5.4.0]undec-7-ene)-mediated route over the classical methods, particularly when the starting compounds are sterically hindered [50]. In this regard, a methanolic solution of PCP ester was allowed to react with a large excess of methanolic hydroxylamine in the presence of DBU for one night. At this stage, the formation of precipitated PCP hydroxamic acids was verified using thin-layer chromatography, with the appearance of the characteristic red spot upon treatment with FeCl_3_ confirming the presence of the hydroxamic acid functionality. Moreover, the formation of the tetra-hydroxamic acid product was confirmed by ^1^H NMR spectroscopy in deuterated DMSO, as evidenced by the disappearance of the characteristic signals of the ethyl group and an upfield shift of 0.27 ppm for the O–CH_2_ protons compared to the spectrum of the PCP ester. However, signals corresponding to DBU at 1.5, 2.3, and 3.1 ppm were still observed (Supporting Information File 1, Figure S7) despite several ultrasonic washings with aqueous ammonium chloride, as previously described by Verma et al. [51]. At this stage, chromatographic techniques, crystallization, and successive washing steps all failed to yield the pure compound due to the persistent presence of DBU.

Additionally, the use of a small amount of solid KCN in aqueous hydroxylamine has been reported for the solution-phase hydroxylamination of esters previously described by Ho et al. [52]. This study demonstrated that the extent of ester conversion and the formation of carboxylic acid by-products vary markedly with the structure of the ester substrate. When this method was used, the ^1^H NMR spectra of the products revealed residual peaks corresponding to unreacted ester (Supporting Information File 1, Figure S8). This observation is consistent with the substrate-dependent behavior reported in that study, highlighting that the reaction outcome is strongly influenced by the nature of the ester substrate. As a result, this synthetic route was discontinued.

**Figure 1:**
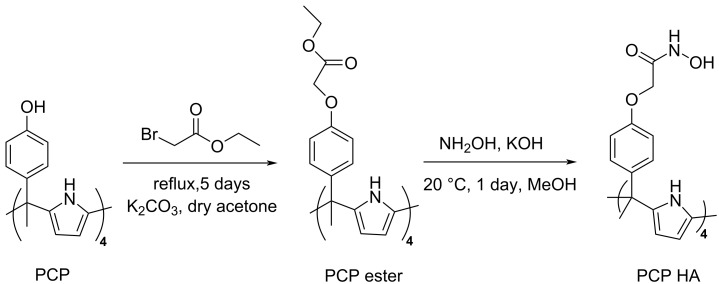
Synthetic route of PCP HA.

Another straightforward method for the synthesis of hydroxamic acids from esters was reported in 1994 by Hutchinson et al., for the synthesis of calixarene tetrahydroxamates from calixarene tetraethylacetate [53]. In the original procedure, KOH was added at −5 °C, and the mixture was stirred for 5 hours at this temperature, followed by 5 days of stirring at room temperature. In our hands, both the addition of KOH and stirring were performed entirely at room temperature, and ^1^H NMR and HRMS monitoring indicated that the reaction reached completion within one day. While some studies have reported the formation of hydroxamic acids from esters at neutral pH [54], in our study the conversion leading to PCP HA (Figure 1) was observed exclusively under alkaline conditions (pH ≥ 10). This finding agrees with earlier methodologies demonstrating that the generation of free hydroxylamine [55] and consequently hydroxamic acid formation occurs efficiently only in alkaline media [48,56]. Moreover, to prevent the formation of carboxylic acid by-products, methanol was used as the solvent both for the generation of free hydroxylamine and for the subsequent synthesis of PCP HA. The resulting tetrapotassium hydroxamate intermediate was then acidified with a 10% HCl solution, inducing precipitation of the tetrahydroxamic acid form. PCP HA was isolated as a grey solid in high yield (95%).

The structure of PCP HA was fully characterized by ^1^H NMR and ^13^C NMR (DMSO-d_6_) as well as by HRMS (ESI^+^) (Figures S3–S6, and S9 in Supporting Information File 1). In the ^1^H NMR spectrum, two sets of singlets at 4.41 and 4.76 ppm (CH_2_) were attributed to the –O=C–CH_2_–O– groups of the *E*/*Z* isomers of the hydroxamic acid moieties, in agreement with previous reports (Supporting Information File 1, Figure S3) [57]. Additionally, two pairs of singlets at 8.97, 9.32 and 10.20, 10.93 ppm (2H total) were assigned to the hydroxy (–OH) and amide (–NH) protons of the hydroxamic acid groups (Supporting Information File 1, Figure S4). The disappearance of these resonances in the 9.0–11.0 ppm region upon D_2_O exchange confirmed these assignments (Supporting Information File 1, Figure S6). Finally, characteristic resonances at 1.76, 5.95, and 8.98 ppm, together with the two doublets at 6.85 ppm, were attributed to the PCP core (Supporting Information File 1, Figure S3). The HRMS (ESI^+^) spectrum exhibited a major peak at *m/z* = 1033.4081 ([M + H]^+^) (Supporting Information File 1, Figure S9), consistent with the expected molecular formula and confirming complete conversion of the PCP ester precursor into the corresponding hydroxamic acid, with no detectable acid-derived by-products. Solubility tests revealed that PCP HA is soluble exclusively in highly polar aprotic solvents (DMSO, DMF) and remains completely insoluble in other common organic solvents (chloroform, ether, etc.) and water. The absence of ligand leaching into water was confirmed by depositing a drop of the aqueous supernatant on a TLC plate, which showed no formation of the characteristic red iron-hydroxamate complex upon treatment with FeCl_3_. These physicochemical properties precluded standard liquid–liquid extraction but were suitable for a solid–liquid approach as previously described for calixarenes [58].

### Extraction experiments

In this study, two parameters that can influence the solid-liquid extraction efficiency of PCP-HA toward uranyl, namely pH and ligand-to-metal ratio, were studied. To ensure comparison between extraction experiments, parameters like temperature, shaking time and volume of uranyl solution used were kept constant. In a typical experiment, 20 mL of a 1 mM aqueous uranyl acetate solution was adjusted to the target pH and then added to solid PCP HA. The solution was shaken at a fixed temperature of 25 °C for 4 h. This time was estimated to be sufficient to reach equilibrium. The heterogeneous solution was centrifuged and the aqueous layer was filtered prior to gamma spectroscopy analysis. Uranium activity concentrations before and after extraction were determined, and extraction efficiencies were calculated from the decrease in activity concentration. It is important to note that the extraction efficiency of PCP HA was evaluated based solely on the quantification of ^238^U. Uranium is a naturally occurring radionuclide that exists predominantly as a mixture of three isotopes: ^238^U, ^235^U and ^234^U. Among these, ^238^U is by far the most abundant, accounting for 99.28 % by mass, followed by ^235^U (0.72 %) and ^234^U (0.0058 %). Although these isotopes possess identical chemical properties, they differ in their radioactive characteristics [59]. Since the extraction experiments depend on the chemical behavior of uranium rather than its radiological properties, any of these isotopes could, in principle, be used. Two main factors justify the choice of ^238^U: its natural abundance and its reliable detectability by gamma spectroscopy, in contrast to ^235^U, which exhibits weak gamma emission intensity [60].

The uncertainties reported have been separated into contributions from the gamma-spectrometric measurements, σ(A_0_) and σ(A_1_), and from the calculations performed to derive the extraction efficiencies’ uncertainty values, σ(% E) and σ(% E_mean_). Measurement uncertainties reflect counting statistics, detector calibration, sample geometry, and matrix effects, while calculation uncertainties account for the propagation of errors during the efficiency computation [61–62]. The uncertainty of the % E was calculated using the law of propagation of uncertainty which accounts for the variability of individual replicates [63]. The reported mean extraction efficiency (% E_mean_) represents the weighted mean of the replicate measurements, with weights inversely proportional to the variance of each replicate [64]. The weights and the equations used in the calculation are provided in Supporting Information File 1, Tables S1–S4.

### Effect of pH

**Figure 2:**
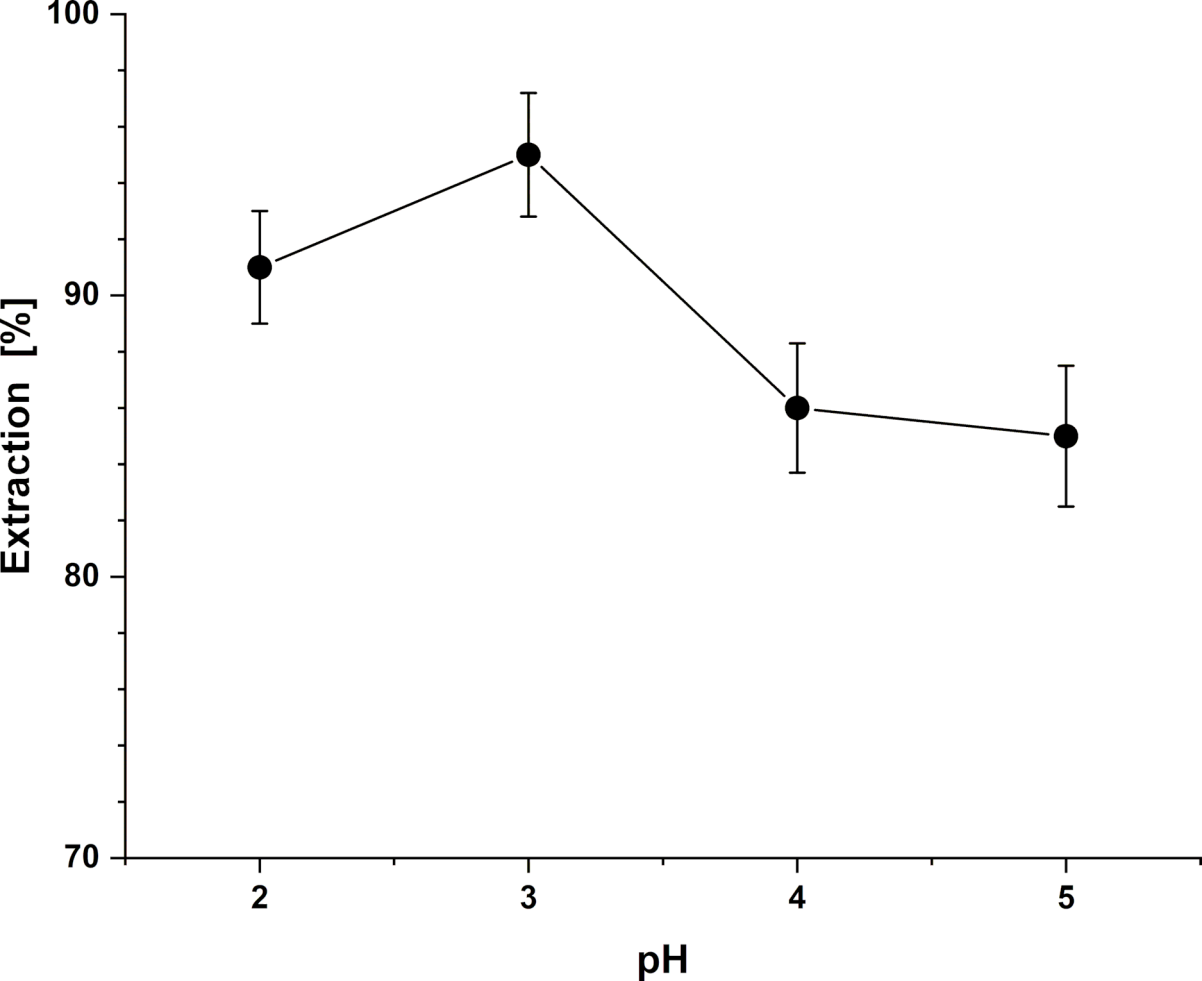
Effect of pH on the mean uranyl extraction efficiency (% E_mean_) of PCP HA.

To gain insight into the binding behavior of PCP HA toward uranyl, the effect of pH on the extraction efficiency was evaluated, as variations in pH can significantly influence metal–ligand complexation equilibria [65]. The results of this experiment are summarized in Table 1. The pH range of 2–5 was specifically chosen based on two considerations: first to avoid PCP HA degradation observed during its synthesis at pH values below 2; and second to prevent the precipitation of uranyl hydroxide solution at pH values above 5 [66]. PCP HA exhibited consistently high extraction efficiencies, 83–95%, across the studied pH range (Table 1), indicating that it maintains strong affinity toward uranyl under moderately acidic conditions. In particular, high extraction efficiencies were observed at pH 2–3, reaching up to 95% at pH 3 (Figure 2). Although a slight increase is observed between pH 2 and pH 3, the extraction efficiencies remain overall comparable within experimental uncertainty, suggesting that uranyl uptake is already near maximal in this acidic region.

**Table 1:** Effect of pH on uranyl extraction by PCP HA.^a^

pH	Replicate	A_0_ (Bq/Kg)	σ(A_0_)	A_1_ (Bq/Kg)	σ(A_1_)	Extraction efficiency (%)	σ(% E)

5	1	2856	362	406	54	86	2.7
	2	2756	367	476	97	83	6.4
4	1	2683	354	399	52	85	2.7
	2	2601	329	287	59	89	4.4
3	1	2525	516	144	31	94	1.8
	2	2611	533	128	28	95	1.6
2	1	2561	518	244	50	90	2.8
	2	2630	532	242	52	91	2.9

^a^A₀ and A₁ represent the activity concentrations of ^238^U in the uranyl acetate working solution before and after extraction, respectively. σ(A₀) and σ(A₁) denote the uncertainties associated with the measured activity concentrations determined by gamma spectroscopy. σ(% E) represents the uncertainties of the individual extraction efficiencies. Extractions were carried out at 25 °C using aqueous uranyl acetate solutions of 1 mM, with the pH being adjusted with 0.1 M HCl. PCP HA was added at a ligand-to-metal molar ratio of 1:1. Samples were equilibrated for 4 h under constant agitation before phase separation and activity measurements.

This behavior is consistent with previous studies in which ligands displayed high uranyl uptake within an optimal pH window. For instance, calix[4]arene-based 8-hydroxyquinoline ligands showed nearly quantitative extraction of uranyl between pH 4 and 9, with efficiency decreasing under more acidic conditions [67]. Likewise, phosphoramide-functionalized magnetic nanoparticles showed high uranyl adsorption (80–95%) between pH 4 and 8, with reduced uptake outside this range [68].

Although hydroxamic acids typically have p*K*_a_ values of 7–9 [69], supramolecular systems bearing these groups do not exhibit uniform uranyl extraction behavior under identical pH conditions. Consequently, some systems achieve maximum extraction under weakly acidic conditions, while others perform better at higher pH values. For instance, an octa-functionalized calix[4]resorcinarenehydroxamic acid exhibits quantitative extraction of uranyl into ethyl acetate solution at pH value of 8 [34]. On the other hand, calix[4]arenes and calix[6]arenes functionalized with hydroxamic acid groups show a marked increase in uranyl extractability from aqueous solution into chloroform at pH 3–4, reaching nearly 100% extractability at pH 5. The enhanced extraction efficiency observed in the acidic pH region has been attributed to the metal-ion-assisted deprotonation. The coordination of the hydroxamic acid groups to the uranyl cation stabilizes the deprotonated hydroxamate form, effectively lowering the apparent p*K*_a_ and enabling strong binding even at low pH [33]. It is important to mention that the formation of the stable 5-membered chelate between the uranyl ion and the Z-isomer of hydroxamic acid effectively shifts the *Z*/*E* equilibrium [70] towards the *Z*-conformation through a metal-induced fit mechanism [71–72]. Consistent with known hydroxamate coordination chemistry, coordination-induced deprotonation of the hydroxamic acid at low pH provides the conditions required for effective uranyl complexation in our system.

### Effect of ligand–metal molar ratio

To further understand the complexation behavior of PCP HA toward uranyl, the effect of the ligand-to-metal molar ratio on the extraction efficiency was investigated. Although the previous experiment revealed maximum extraction at pH values of 2 and 3, the investigation of the ligand-to-metal molar ratio was conducted at pH 4. This adjustment was made to allow a clearer assessment of the molar ratio effect. At pH 3, a 1:1 ratio of PCP HA to uranyl already yielded about 95% extraction, leaving little sensitivity to detect variations at higher ligand mass. By performing the experiment at pH 4, where the extraction efficiency was approximately 86%, the system remained sufficiently responsive to changes in ligand mass.

**Figure 3:**
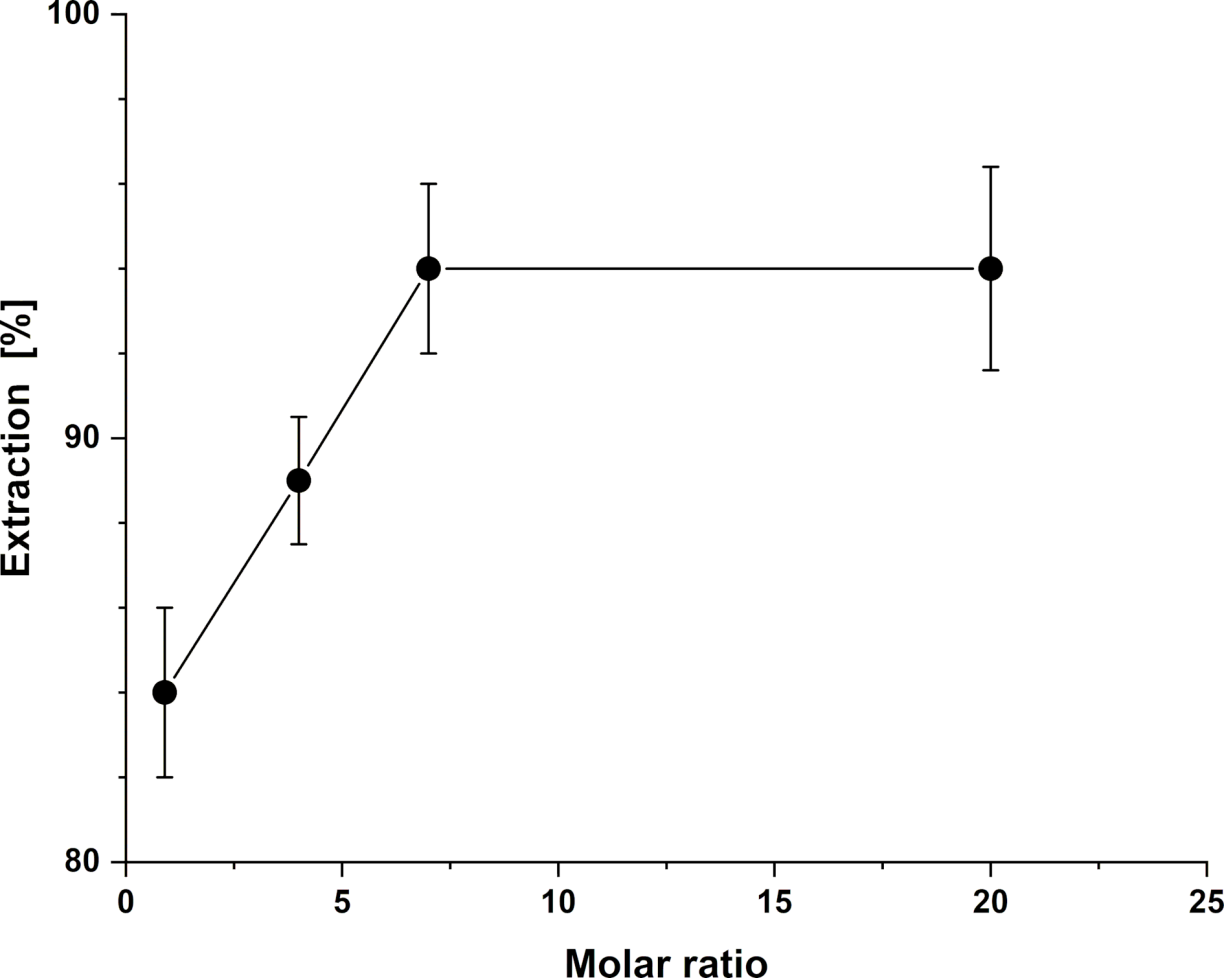
Effect of ligand-to-metal molar ratio on the mean uranyl extraction efficiency (% E_mean_) of PCP HA.

Starting from a ligand-to-metal molar ratio of 0.9:1, the extraction efficiency increased steadily with increasing ligand mass, reaching a maximum of about 94% at a ratio of 7:1 (Table 2). Beyond this point, the ligand mass was increased to achieve a 20:1 ratio, yet no further improvement in extraction efficiency was observed (Figure 3). This finding indicates that at pH 4, a ligand-to-metal molar ratio of 7:1 is sufficient to ensure nearly complete complexation of uranyl. This apparent excess reflects the heterogeneous nature of the system. Since a significant portion of the hydrophilic groups responsible complexation with uranium [58] is likely buried within the solid bulk and thus inaccessible, a high nominal ratio is necessary to provide a sufficient number of available coordination sites at the water–solid interface to quantitatively capture the uranyl ions [73].

**Table 2:** Effect of ligand–metal ratio on uranyl extraction by PCP HA.

Ligand–metal molar ratio	Replicate	A_0_ (Bq/Kg)	σ(A_0_)	A_1_ (Bq/Kg)	σ(A_1_)	Extraction efficiency (%)	σ(% E)

0.9:1	1	1535	166	224	28	85	
	2	1576	177	277	31	82	2.8
4:1	1	1520	308	164	21	89	1.7
	2	1524	173	147	30	90	4.2
7:1	1	1577	178	100	21	94	2.8
	2	1574	169	94	20	94	2.8
20:1	1	1476	174	95	23	94	3.7
	2	1509	163	94	21	94	3.2

The observed efficiency depends not only on ligand mass but also on the intrinsic coordination preferences with uranyl. In aqueous solution, hexavalent uranium, the most dominant oxidation state, exists predominantly as the linear uranyl ion UO_2_^2+^. In this ion, two oxo ligands occupy axial positions while the equatorial plane accommodates four to six donor atoms [74–75]. Complexes of UO_2_^2+^ often adopt either a pseudoplanar pentacoordinate or hexacoordinate structure, as shown by X-ray crystallographic studies. Consequently, to bind UO_2_^2+^ effectively, a ligand must present donor atoms positioned to match the uranyl equatorial coordination sites [76].

Hydroxamic acids act as bidentate ligands, with each functional unit offering two donor atoms, a carbonyl oxygen and a hydroxy oxygen, that can simultaneously coordinate to a uranyl ion [77]. In supramolecular systems, not all hydroxamic acid groups necessarily bind uranyl ions, as steric hindrance and site accessibility can limit coordination to a subset of available sites [78]. A relevant example is a calix[6]arene functionalized with three hydroxamic acid groups, where a theoretical study using density functional theory (DFT) calculations showed that the complex with the uranyl is most stabilized when only two of the three hydroxamic acid units participate in binding [78]. This concept of partial participation and cooperative interactions between hydroxamic acid units may also be relevant in the PCP HA system, where not all functional groups are necessarily involved in uranyl binding.

## Conclusion

In this study, we have efficiently synthesized PCP HA, a phenoxycalix[4]pyrrole scaffold functionalized with four hydroxamic acid groups, and demonstrated its uranyl extraction potential. Solid–liquid extraction studies showed that it removes up to 95% of uranyl at pH 3. Subsequent investigation at pH 4 revealed that a 7:1 ligand-to-metal molar ratio is sufficient to achieve near-quantitative uranium removal (≈94%). The strong coordination provided by the hydroxamic acid groups within the pre-organized cavity likely underlies its high extraction efficiency. These results highlight PCP HA as a promising supramolecular platform for uranyl removal and open the door for its application in the remediation of uranium-contaminated environmental samples. Future work will focus on the covalent anchoring of this macrocycle onto solid matrixes (polymer or silica). The development of such functionalized material is intended to allow for implementation in continuous flow filtration systems for industrial effluent remediation or for the challenging task of uranium extraction from seawater [79].

## Experimental

Detailed synthetic procedures, compound characterization (^1^H and ^13^C NMR, HRMS), and uranyl extraction protocols, including pH and ligand-to-metal ratio studies, are described in Supporting Information File 1.

## Supporting Information

File 1 Experimental part.

## Data Availability

All data that supports the findings of this study is available in the published article and/or the supporting information of this article.
