# Supplementary material for: Synthesis and uranyl(VI) extraction performance of a calix[4]pyrrole–tetrahydroxamic acid receptor
Source: Beilstein J Org Chem. 2026 Mar 18;22:486–94. doi: 10.3762/bjoc.22.36 (PMC13033896; doi:10.3762/bjoc.22.36)
Supplement: File 1 — Experimental part. [file Beilstein_J_Org_Chem-22-486-s001.pdf]

BEILSTEIN JOURNAL OF ORGANIC CHEMISTRY

Supporting Information

for

Synthesis and uranyl(VI) extraction performance of a calix[4]pyrrole–tetrahydroxamic acid receptor

Sara Karnib, Rana Baydoun, Wissam Zaidan, Nancy AlHaddad, Omar El Samad, Bilal Nsouli, Francine Cazier-Dennin and Pierre-Edouard Danjou

Beilstein J. Org. Chem. **2026,** *22,* 486–494. doi:10.3762/bjoc.22.36

Experimental part

License and Terms: This is a supporting information file under the terms of the Creative Commons Attribution License (https://creativecommons.org/licenses/by/4.0). Please note that the reuse, redistribution and reproduction in particular requires that the author(s) and source are credited and that individual graphics may be subject to special legal provisions.
The license is subject to the *Beilstein Journal of Organic Chemistry* terms and conditions: (https://www.beilstein-journals.org/bjoc/terms)

Table of contents

1. General information S2

2. Synthetic procedure and characterization data S3,4

3. NMR spectra S5–9

4. Extraction experiments procedure S9,10

5. Calculation of the uncertainty and the mean percentage of extraction values S10–13

6. References S13

1. General Information

All chemicals and reagents used for synthesis were purchased from commercial suppliers and used without any further purification. Synthesis of PCP ester derivative (PCP E) was performed under argon atmosphere.

Nuclear magnetic resonance (NMR) spectra were recorded at 25 °C using a Bruker Avance III Spectrometer operating at 400 MHz and 100 MHz for ^{1}H NMR and ^{13}C NMR respectively. Samples were prepared in deuterated DMSO-d_6. Chemical shifts are reported in parts per million (ppm) relative to residual solvent peak (^{1}H NMR, DMSO = 2.50 ppm; ^{13}C NMR, DMSO = 39.52 ppm). Multiplicities are designated as s (singlet), d (doublet), t (triplet), m (multiplet). Coupling constants (J) are given in hertz (Hz). Peaks labeled with an asterisk (*) in the spectra correspond to residual solvent signals and/or impurities and are not attributed to the target molecule.

High-resolution mass spectra were acquired on a SCIEX X500 QTOF operated in positive ion mode. Samples were prepared in DMSO, diluted with methanol, and directly injected with methanol in the presence of 0.1% formic acid as the mobile phase. The instrument was operated with a spray voltage of 5500 V, a declustering potential of 80 V, and a collision energy of 10 V.

Gamma spectrometry measurements were conducted at the Gamma Spectroscopy Laboratory at the Lebanese Atomic Energy Commission-LAEC using gamma spectrometers with p-type coaxial High Purity Germanium detectors (HPGe). The spectra were analyzed offline using Genie 2000 software from Canberra Version 3.1b. Uranium-238 was determined from the gamma line of its daughter Th-234.

2. Synthetic Procedures and Characterization data

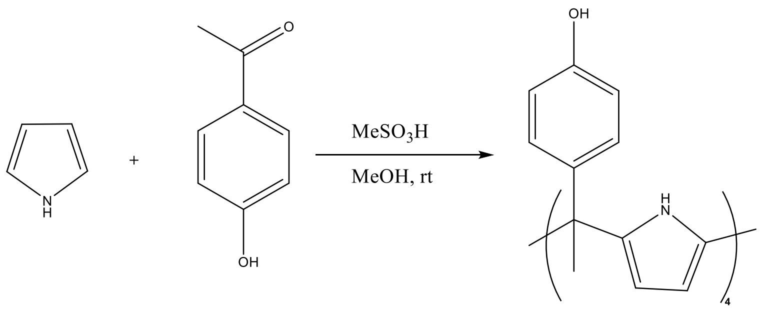

1

PCP

2.1. Synthesis of *meso*-tetra(phenoxy)calix[4]pyrrole (PCP): PCP was synthesized through the acid catalyzed condensation of pyrrole with 4-hydroxyacetophenone (**1**) based on a previously described procedure [1]. PCP was obtained as either a pink, brown or beige powder in a 47% yield.

^{1}H NMR spectrum (400 MHz, DMSO-d6): δ 9.42 (s, 4 H, NH), 9.30 (s, 4H, OH), 6.67 (d, *J*=8.7 Hz, ,8H, Ar), 6.61 (d, *J*=8.7 Hz, 8H, Ar), 5.92 (d, *J*= 2.4 Hz, 8H, pyrrolic CH), 1.70 (s, 12H, CH_3).

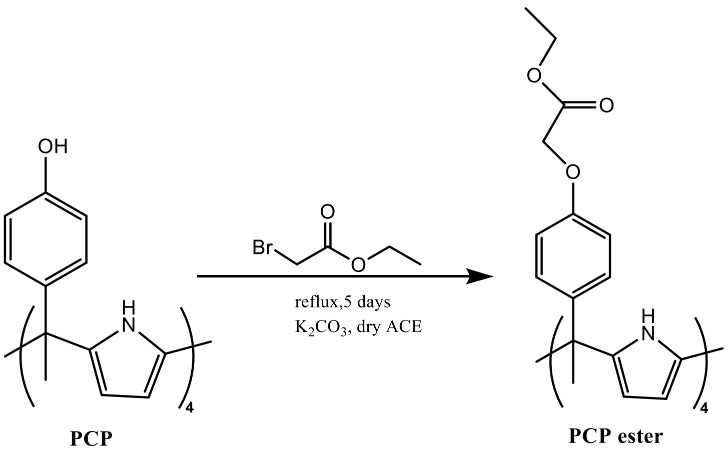

PCP

PCP ester

2.2. Synthesis of *meso*-tetra[4-(ethoxycarbonylmethoxy)phenyl]calix[4]pyrrole (PCP E): The ester derivative was synthesized through an O-alkylation reaction of PCP and ethyl bromoacetate in dry ACE/ K_2CO_3 under Argon atmosphere according to a previously described procedure [2]. PCP E was obtained as a white powder in 85% yield.

^{1}H NMR spectrum (400 MHz, DMSO-d6): δ 9.45 (s, 4H, NH), 6.86 (d, J=8.8 Hz, 8H, Ar), 6.77 (d, J=8.8 Hz, 8H, Ar), 5.97 (s, 8H, pyrrolic CH), 4.69 (s, 8H, O-CH_2-CO), 4.15 (q, J=7.1 Hz, 8H, O-$\underline{CH_2}$-CH_3), 1.73 (s, 12H, CH_3), 1.21 (t, 12H, O-CH_2-$\underline{CH_3}$).

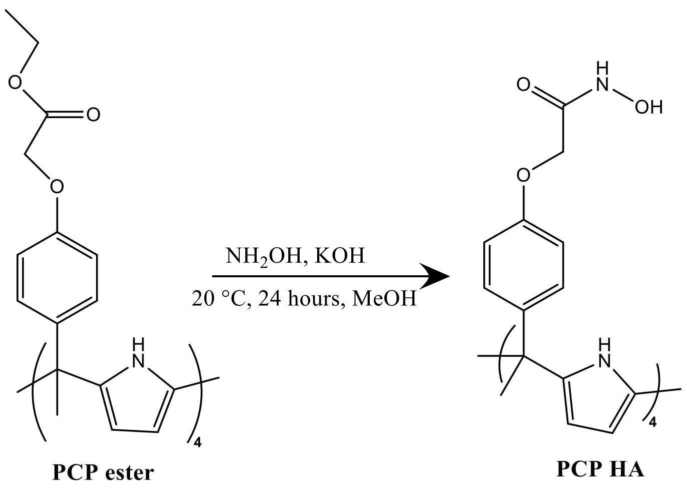

2.3. Synthesis of *meso*-[tetra(hydroxamic acid)phenyl]calix[4]pyrrole (PCP HA): The hydroxamic acid derivative was synthesized according to a literature protocol originally reported for a calix[4]arenes [3]. To a solution of compound PCP E (200 mg, 0.184 mmol) in 5 mL methanol was added hydroxylamine (22.11 mmol, 120 equiv), freshly prepared as described below. Potassium hydroxide (0.50 g, 8.5 mmol, 46 equiv) was then introduced, and the reaction mixture was stirred at 20 °C for 24 h. The mixture was subsequently acidified with 10% HCl to adjust the pH to 1–2, followed by centrifugation. The resulting precipitate was washed four times with water, then dried under high vacuum to afford the product as a white to grey solid in a 95% yield.

Preparation of hydroxylamine: Hydroxylamine was generated following the method recommended by Reddy et al. [4], based on the procedure reported by Devlin et al. [5], with minor modifications. Hydroxylamine hydrochloride (1.53 g, 22.11 mmol) was dissolved in 15 mL of methanol and added to a solution of potassium hydroxide (1.42 g, 25.42 mmol) in 5 mL of methanol. The mixture was stirred at 20 °C for 15 min, and the precipitated potassium chloride was removed by centrifugation. The resulting supernatant, containing hydroxylamine, was directly used in the subsequent reaction with the ester.

^{1}H NMR spectrum (400 MHz, DMSO-d6): δ 10.93 (s, 4H, NH-OH, Z-isomer), 10.20 (s, 4H, NH-OH, E-isomer) 9.45 (s, 4H, NH), 9.32 (s, 4H, OH, Z-isomer), 8.97 (s, 4H, OH, E-isomer), 6.88 (d, J=7.3, 8H, Ar), 6.79 (d, J=7.4, 8H, Ar), 5.94 (s, 8H, pyrrolic CH), 4.76 (s, 4H, O-CH_2, Z-isomer), 4.41 (s, 4H, O-CH_2, E-isomer), 1.74 (s, 12H, CH_3).

^{13}C NMR (100 MHz, DMSO-d6): δ 164.89 (C=O), 157.05 (C-O, Ar), 143.29 (C-C, Ar), 137.77 (Cα, pyrrole), 128.29 (CH, Ar), 114.86 (CH, Ar), 105.0 (Cß, pyrrole), 66.27 (O-CH_2), 43.93 ($C(CH_3)_3$), 31.74 (CH_3).

FTIR: 3243 cm^{-1} (NH/OH, hydroxamic and pyrrolic), 1654 cm^{-1} (C=O, hydroxamic).

HRMS (ESI$^+$) m/z [M+H]$^+$ calculated for $C_{56}H_{57}N_8O_{12}^+$ 1033.4090, found 1033.4081 (error = 0.967 ppm)

3. NMR spectra

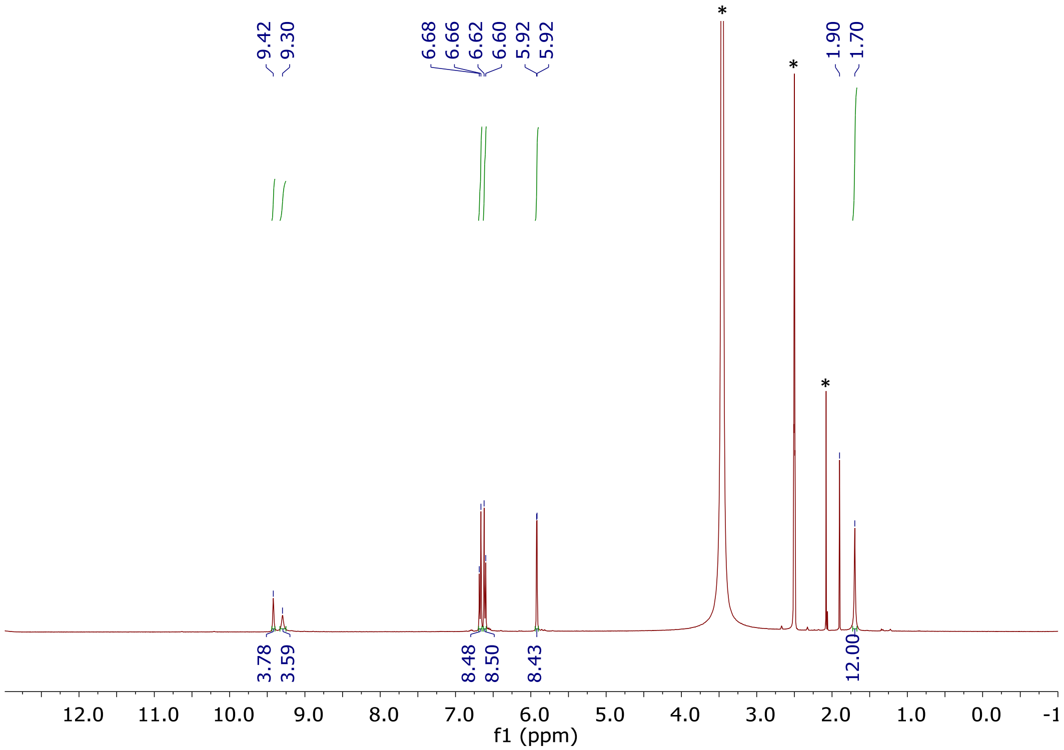

Figure S1. ^{1}H NMR spectrum of PCP (DMSO-d_6)

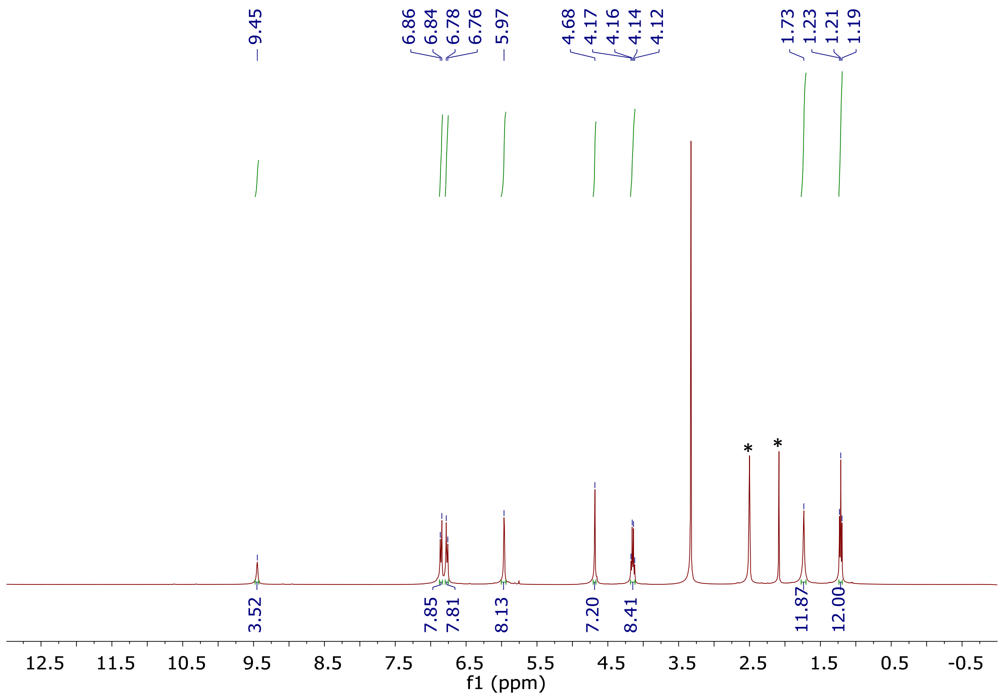

Figure S2. ^{1}H NMR spectrum of PCP E (DMSO-d_6)

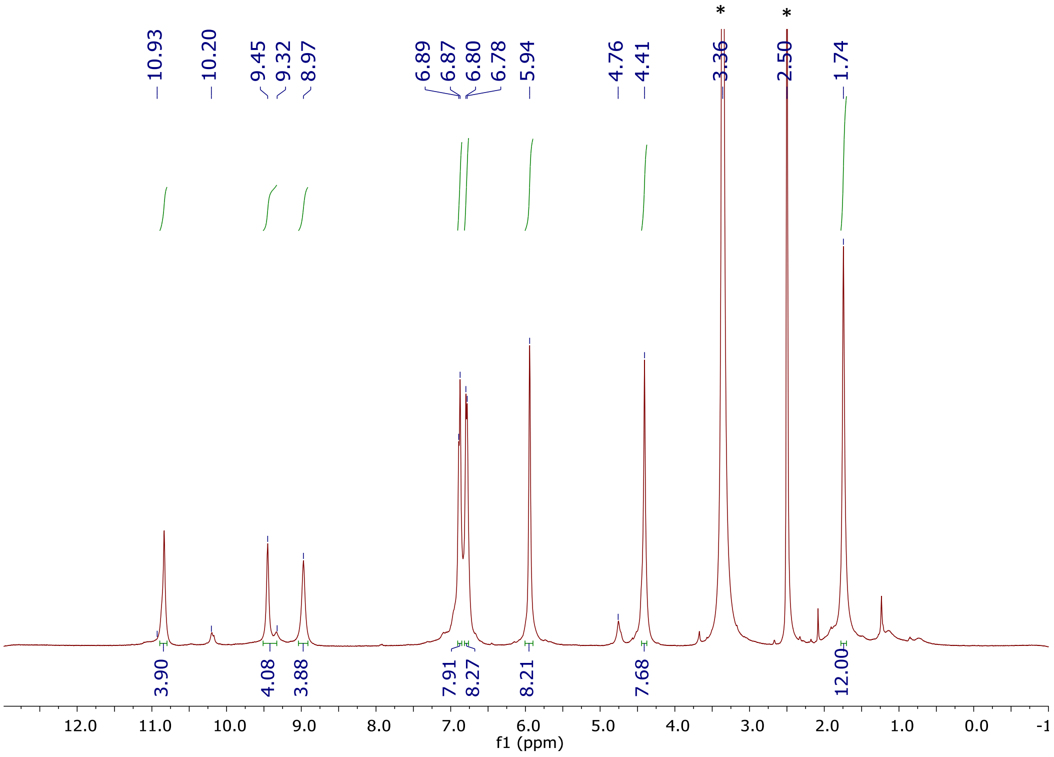

Figure S3. ^{1}H NMR spectrum of PCP HA (DMSO-d_6)

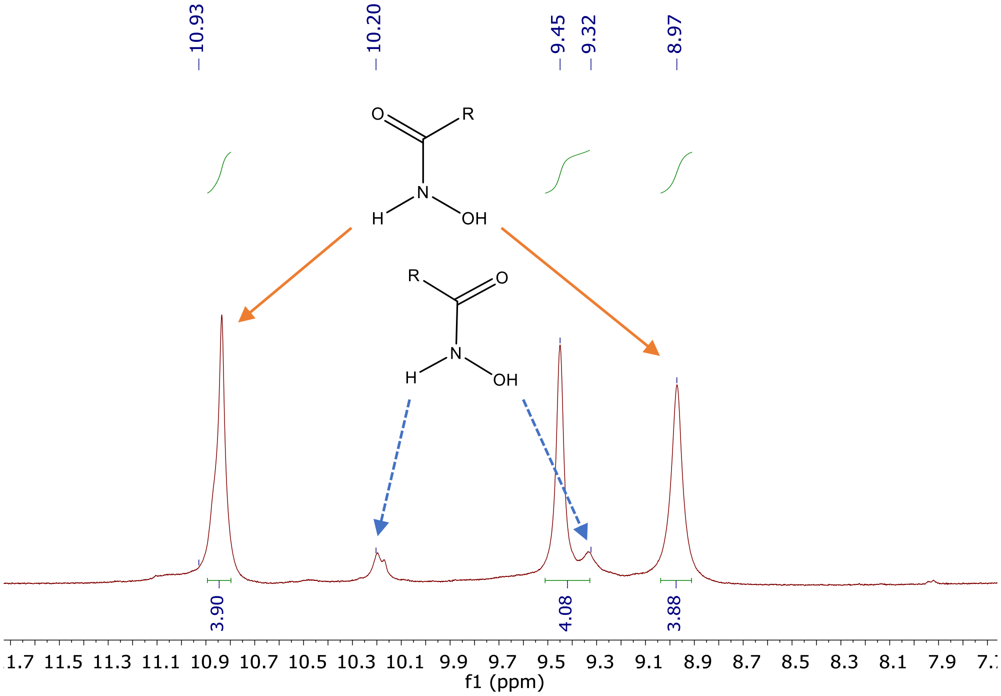

Figure S4. Selected region of the ^{1}H NMR spectrum of PCP HA (DMSO-d_6)

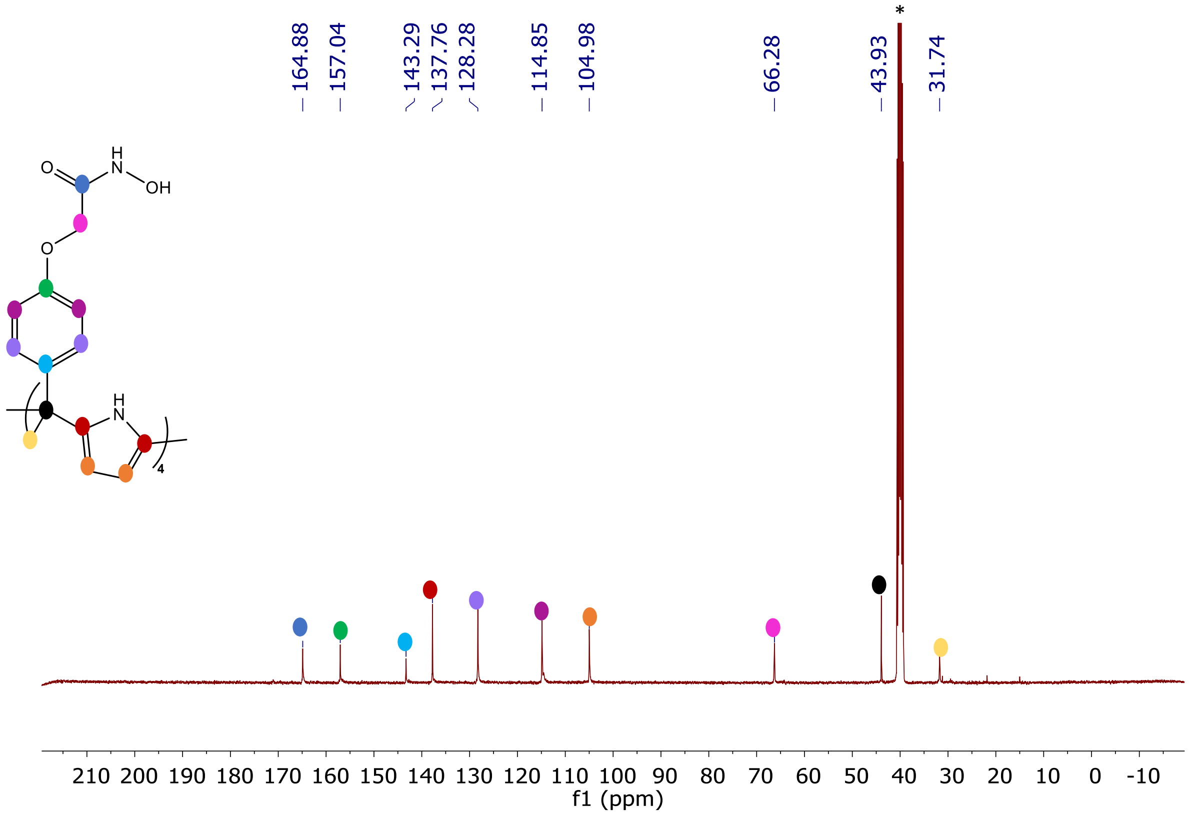

Figure S5. ^{13}C NMR spectrum of PCP HA (DMSO-d_6)

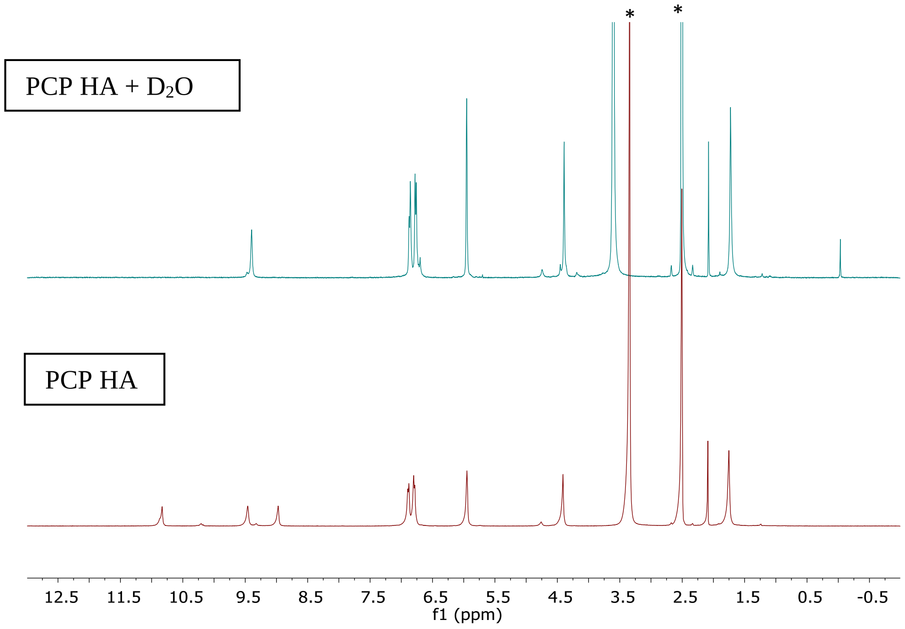

Figure S6. ^{1}H NMR D_2O exchange experiment of PCP HA (DMSO-d_6)

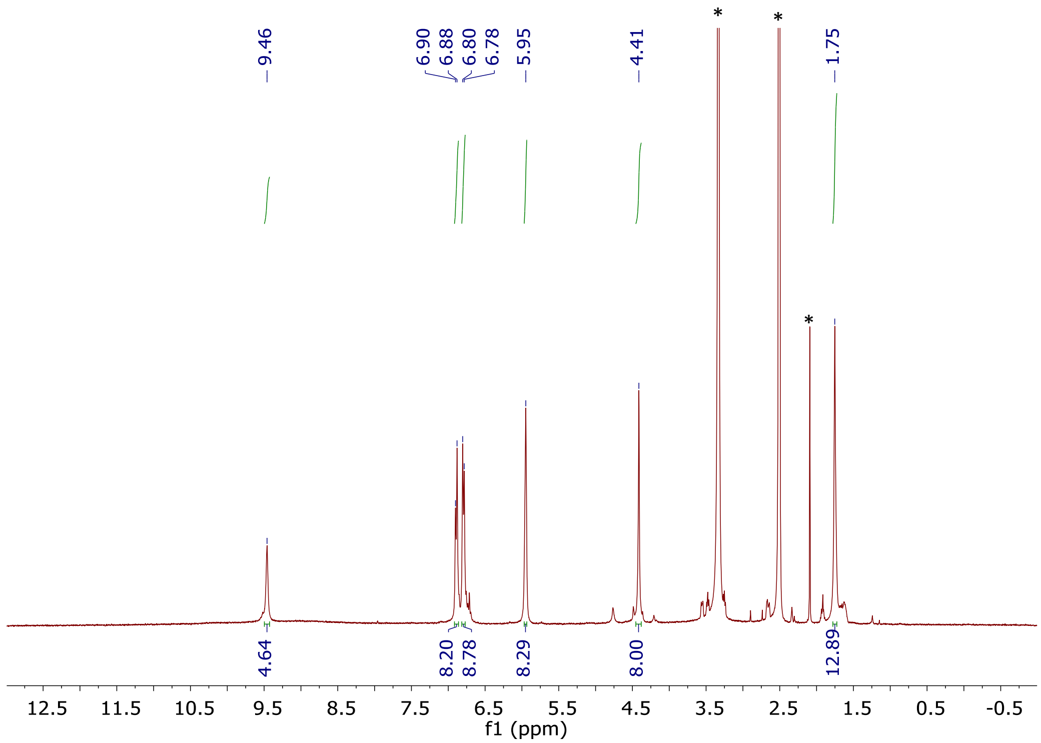

Figure S7. ^{1}H NMR spectrum of PCP HA using DBU approach (DMSO-d_6)

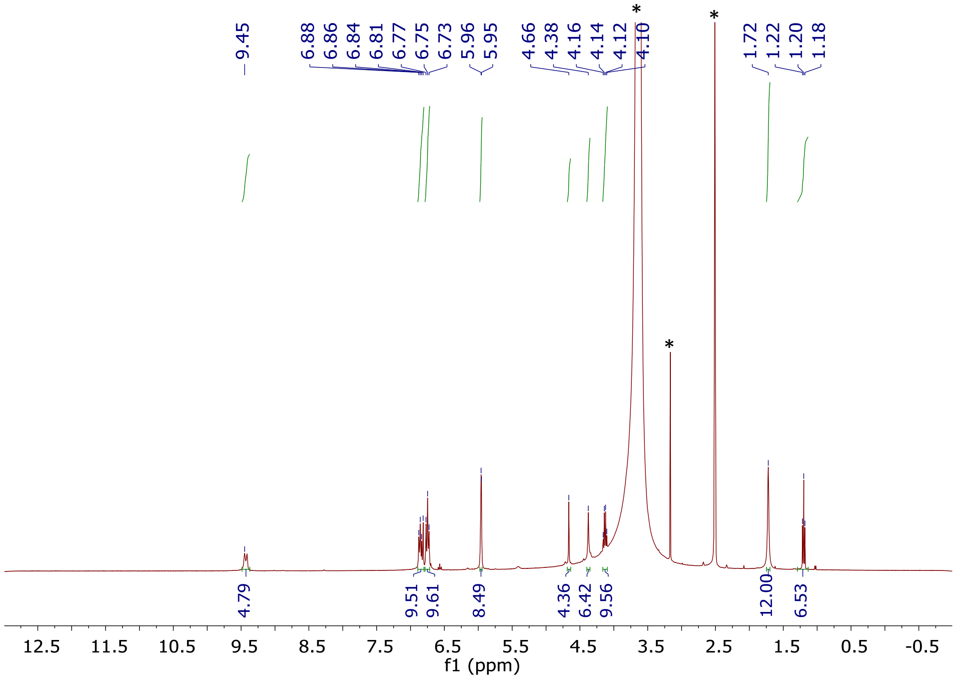

Figure S8. ^{1}H NMR spectrum of PCP HA using KCN approach (DMSO-d_6)

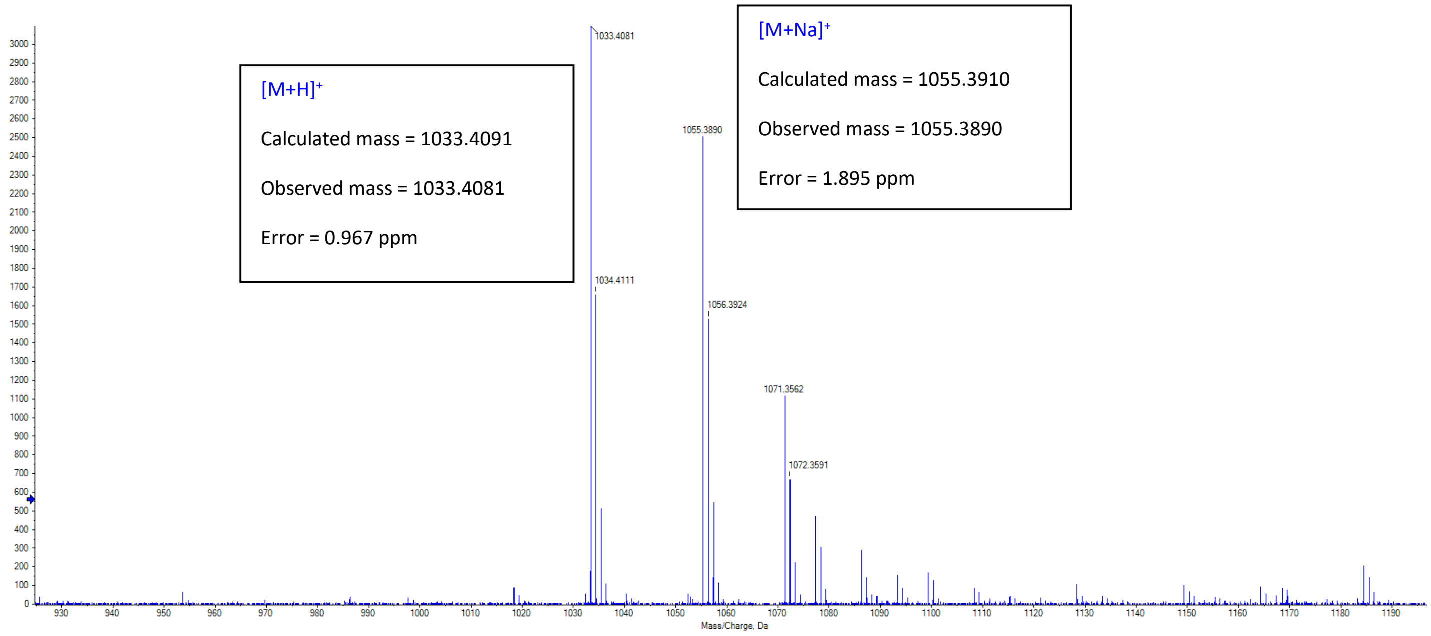

Figure S9. HRMS spectrum of PCP HA

4. Extraction experiments procedure

4.1. General extraction procedure

Working solutions of uranyl acetate (1mM) were prepared by dilution of a stock solution with an activity concentration of 662 ± 83 KBq/Kg using deionized water. The pH of the solutions was adjusted with 0.1 M HCl, when required.

In all experiments, 20 mL of uranyl acetate working solutions were added to the appropriate mass of PCP HA and the suspension was shaken for 4 h at 25 °C. After equilibration, the mixtures were centrifuged at 3000 rpm for 5 minutes, and the aqueous layer was separated from the powder by filtration. The activity concentration of uranyl acetate solutions was measured before and after extraction using gamma spectrometry. The extraction efficiency was calculated according to the following equation:

$$\%\,Extraction = \frac{(A0 - A1)}{A0} \times 100$$

Here, A0 and A1 represent uranium activity concentrations in the working solutions before and after extraction, respectively. All experiments were performed in duplicate.

4.2. Effect of pH

To examine the effect of acidity on the extraction process, solutions of pH 2, 3, 4 and 5 were prepared by careful adjustment of the pH of uranyl acetate working solution. A constant ligand to metal molar ratio of 1:1 was maintained in this experiment. The extraction was performed as described above.

4.3. Effect of Ligand to metal molar ratio

To evaluate the effect of ligand concentration on the extraction, varying masses of PCP HA were mixed with 20 mL uranyl acetate working solutions (1 mM, pH = 4). The ligand to metal ratio investigated ranged from 0.9:1 to 14:1. Extractions were carried out as described in the general procedure above.

5. Calculation of the uncertainty and the mean percentage of extraction values

Using the law of propagation of uncertainty,[6] the uncertainty of the percentage of extraction ($\sigma_{\%E}$) of each replicate was calculated from the measured uncertainty values of A_0 (σ_0) and A_1 (σ_1) according to the following equation:

$$\sigma\ (\%E) = [\, f \times \sqrt{\left(\frac{\sigma 0}{A0}\right)^2 + \left(\frac{\sigma 1}{A1}\right)^2}\,] \times 100$$

Where $f = \frac{\sigma 1}{\sigma 0}$

Table S1. Calculated uncertainty values of the percentage of extraction of PCP HA as a function of pH

pH	A0 (Bq/Kg)	σ0	A1 (Bq/Kg)	σ1	% E	f	σ0/A0	σ1/A1	σ %E
5	2856	362	406	54	86	0.149	0.127	0.133	2.7
	2756	367	476	97	83	0.265	0.133	0.204	6.4
4	2683	354	399	52	85	0.147	0.132	0.130	2.7
	2601	329	287	59	89	0.181	0.127	0.207	4.4
3	2525	516	144	31	94	0.060	0.205	0.216	1.8
	2611	533	128	28	95	0.052	0.204	0.218	1.6
2	2561	518	244	50	90	0.097	0.202	0.207	2.8
	2630	532	242	52	91	0.098	0.202	0.215	2.9

Table S2. Calculated uncertainty values of the percentage of extraction of PCP HA as a function of ligand to metal molar ratio

Molar ratio	A0 (Bq/Kg)	σ0	A1 (Bq/Kg)	σ1	% E	f	σ0/A0	σ1/A1	σ %E
**0.9**	1535	166	224	28	85	0.170	0.108	0.126	2.8
	1576	177	277	31	82	0.175	0.112	0.112	2.8
**4**	1520	308	164	21	89	0.069	0.203	0.130	1.7
	1524	173	147	30	90	0.176	0.113	0.208	4.2
**7**	1577	178	100	21	94	0.117	0.113	0.208	2.8
	1574	169	94	20	94	0.118	0.107	0.213	2.8
**20**	1476	174	95	23	94	0.135	0.118	0.247	3.7
	1509	163	94	21	94	0.128	0.108	0.222	3.2

The mean percentage of extraction represents the weighted mean calculated from the percentage extraction values of the individual replicates according to the following equation: [7]

$$\%E_{\text{mean}} = \frac{(\%E1 \times W1) + (\%E2 \times W2)}{W1 + W2}$$

Where, $W1 = \frac{1}{(\sigma 1)2}$ and $W2 = \frac{1}{(\sigma 2)2}$

With σ1 and σ2 corresponding to the uncertainty values of the percentage of extraction of the first and the second replicate, respectively.

The uncertainty of the mean %E ($\sigma_{\%E\text{mean}}$) is calculated according to the following equation: [7]

$$\sigma_{\%E\text{mean}} = \sqrt{\frac{1}{W1 + W2}}$$

Table S3. Calculated mean percentage of extraction values of PCP HA as a function of pH

pH	% E1	σ %E1	W1	% E2	σ %E2	W2	$\%E_{mean}$	$\sigma_{\%E\ mean}$
**5**	86	2.7	0.134	83	6.4	0.024	85	2.5
**4**	85	2.7	0.135	89	4.4	0.052	86	2.3
**3**	94	1.8	0.310	95	1.6	0.411	95	1.2
**2**	90	2.8	0.127	91	2.9	0.119	91	2.0

Table S4. Calculated mean percentage of extraction values of PCP HA as a function of ligand to metal molar ratio

molar ratio	% E1	σ %E1	W1	% E2	σ %E2	W2	$\%E_{mean}$	$\sigma_{\%E\ mean}$
**0.9**	85	2.8	0.126	82	2.8	0.131	84	2.0
**4**	89	1.7	0.365	90	4.2	0.057	89	1.5
**7**	94	2.8	0.131	94	2.8	0.126	94	2.0
**20**	94	3.7	0.073	94	3.2	0.099	94	2.4

6. References

(1) Danil de Namor, A. F.; Shehab, M. *J. Phys. Chem. B* **2005**, *109*, 17440–17444. doi:10.1021/jp0530707

(2) Camiolo, S.; Gale, P. A. *Chem. Commun.* **2000**, No. 13, 1129–1130. doi:10.1039/b003229h

(3) Hutchinson, S.; Kearney, G. A.; Horne, E.; Lynch, B.; Glennon, J. D.; Anthony McKervey, M.; Harris, S. J. *Analytica Chimica Acta* **1994**, *291*, 269–275. doi:10.1016/0003-2670(94)80022-7

(4) Reddy, A. S.; Kumar, M. S.; Reddy, G. R. *Tetrahedron Letters* **2000**, *41*, 6285–6288. doi:10.1016/S0040-4039(00)01058-3

(5) Devlin, J. P.; Ollis, W. D.; Thorpe, J. E. *J. Chem. Soc., Perkin Trans. 1* **1975**, No. 9, 846–848. doi:10.1039/P19750000846

(6) Arnold. D; Debertin. K; Heckel. A; Kanisch. G; Wershofen. H; Wilhelm. C. *Fundamentals of Gamma Spectrometry*; German Federal Ministry for the Environment, Nature Conservation, Nuclear Safety and Consumer Protection (BMUV), 2018

(7) U.S. Environmental Protection Agency. *High Resolution Gamma-Ray Spectrometry Analyses for Normal Operations and Radiological Incident Response*; EPA 402-B-17-001; U.S. Environmental Protection Agency: Washington, D.C., October
